# The association between testosterone and serum soluble klotho in the females: evidence from the NHANES database

**DOI:** 10.3389/fendo.2024.1335611

**Published:** 2024-05-16

**Authors:** Qi Cao, Jiani Zhang, Xiaohu Hao, Siyu Du, Lu Ao, Huili Zhu, Wei Huang

**Affiliations:** ^1^ Department of Reproductive Medical Center, West China Second University Hospital, Sichuan University, Chengdu, Sichuan, China; ^2^ Key Laboratory of Birth Defects and Related Diseases of Women and Children (Sichuan University), Ministry of Education, Sichuan University, Chengdu, Sichuan, China; ^3^ West China School of Medicine, Sichuan University, Chengdu, Sichuan, China; ^4^ Department of Obstetrics and Gynecology, West China Second University Hospital, Sichuan University, Chengdu, China; ^5^ Department of Thoracic Surgery, West China Hospital, Sichuan University, Chengdu, Sichuan, China; ^6^ West China School of Public Health and West China Fourth Hospital, Sichuan University, Chengdu, Sichuan, China

**Keywords:** sex hormones, S-klotho, female, NHANES, testosterone

## Abstract

**Objective:**

This research aimed to elucidate the relationship between testosterone levels and serum soluble klotho (S-klotho) concentrations in females aged 40-79 years using the National Health and Nutrition Examination Survey (NHANES) dataset.

**Design:**

Associations between testosterone and S-klotho were assessed through multivariable linear regression methodologies, spanning nonadjusted, minimally adjusted, and fully adjusted models.

**Settings:**

The investigation was conducted as a cross-sectional analysis utilizing the NHANES database.

**Participants:**

From 20,146 NHANES participants between 2013 and 2016, 2,444 females met the stipulated inclusion and exclusion criteria.

**Results:**

Free androgen index (FAI) showcased a negative correlation with S-klotho levels across all regression models (nonadjusted: β -7.08, 95% CI -13.39- -0.76; minimally adjusted: β -9.73, 95% CI -16.6- -2.84; fully adjusted: β -7.63, 95% CI -14.75-0.51). Conversely, total testosterone did not exhibit significant associations with S-klotho across the models. In the nonadjusted model, estradiol was positively associated with S-klotho concentrations (β 0.14, 95% CI 0.05-0.23), but this significance was not retained in subsequent regression models.

**Conclusion:**

Findings suggest that in U.S. females aged 40-79 years, FAI negatively correlates with S-klotho concentrations, while there is the lack of significant associations for total testosterone and estradiol.

## Introduction

Klotho is a transmembrane protein that is encoded by the *klotho* gene and serves as a coreceptor for fibroblast growth factor 23 (FGF23) (1, 2). Its major expression site is the distal convoluted tubules within the kidney, playing a critical role in multiple essential physiological processes such as inflammation regulation, antioxidation, and senescence prevention (3, 4). Klotho is present in two primary forms: the membrane-bound version and its secreted counterpart. The soluble form, referred to as soluble Klotho (S-klotho), can be identified in various bodily fluids like blood, urine, and cerebrospinal fluid (5, 6). Deficiencies in S-klotho have been implicated in several age-related conditions, including atherosclerosis, cognitive decline, and reduced bone mineral density (7, 8). Notably, overexpression of S-klotho has been shown to prolong the life span of transgenic mice by a remarkable 30% (9).

Androgens are essential in both sexes, governing processes ranging from growth and development to reproduction. Testosterone, a primary steroid hormone, is predominantly synthesized by the gonads and is a standard clinical measure for evaluating androgen levels (10). In females, testosterone is chiefly produced by the ovaries and adrenal glands, while in males, the testis is the primary site of production. Female testosterone levels observe a gradual decline leading up to menopause, post which a sharp drop is evident - an indication of the correlation between reduced testosterone levels and aging (11). Another crucial component in this hormonal framework is the sex hormone–binding globulin (SHBG), predominantly secreted by the liver, with localized secretion observed in organs like the testes, uterus, and brain (12). SHBG exhibits a high binding affinity to testosterone, influencing the proportion of free testosterone in circulation (13). The free androgen index (FAI) is a derived metric, obtained by the ratio of total testosterone to SHBG, multiplied by 100, and serves as an indicator of abnormal androgen status (14).

Prior research has delved into the interplay between Klotho and sex hormones. A case-control study from China found a correlation between reduced Klotho expression and declining ovarian reserves during reproductive aging (15). From the animal model perspective, it was observed that a deficiency in estradiol potentially augmented Klotho protein expression at the genomic level, subsequently leading to increased urinary calcium excretion (16). A separate study focusing on the U.S. male population discerned an increasing trend in Klotho levels with rising levels of testosterone, estradiol, and SHBG, based on NHANES data (17). However, existing literature offers limited insights regarding the relationship between androgens and S-klotho in an expansive female cohort. This study addresses significant research gaps concerning the potential relationships between S-klotho concentrations and testosterone levels, particularly focusing on female-specific data, utilizing the NHANES dataset as a foundation.

## Methods

### Study design and participants

**Figure 1:**
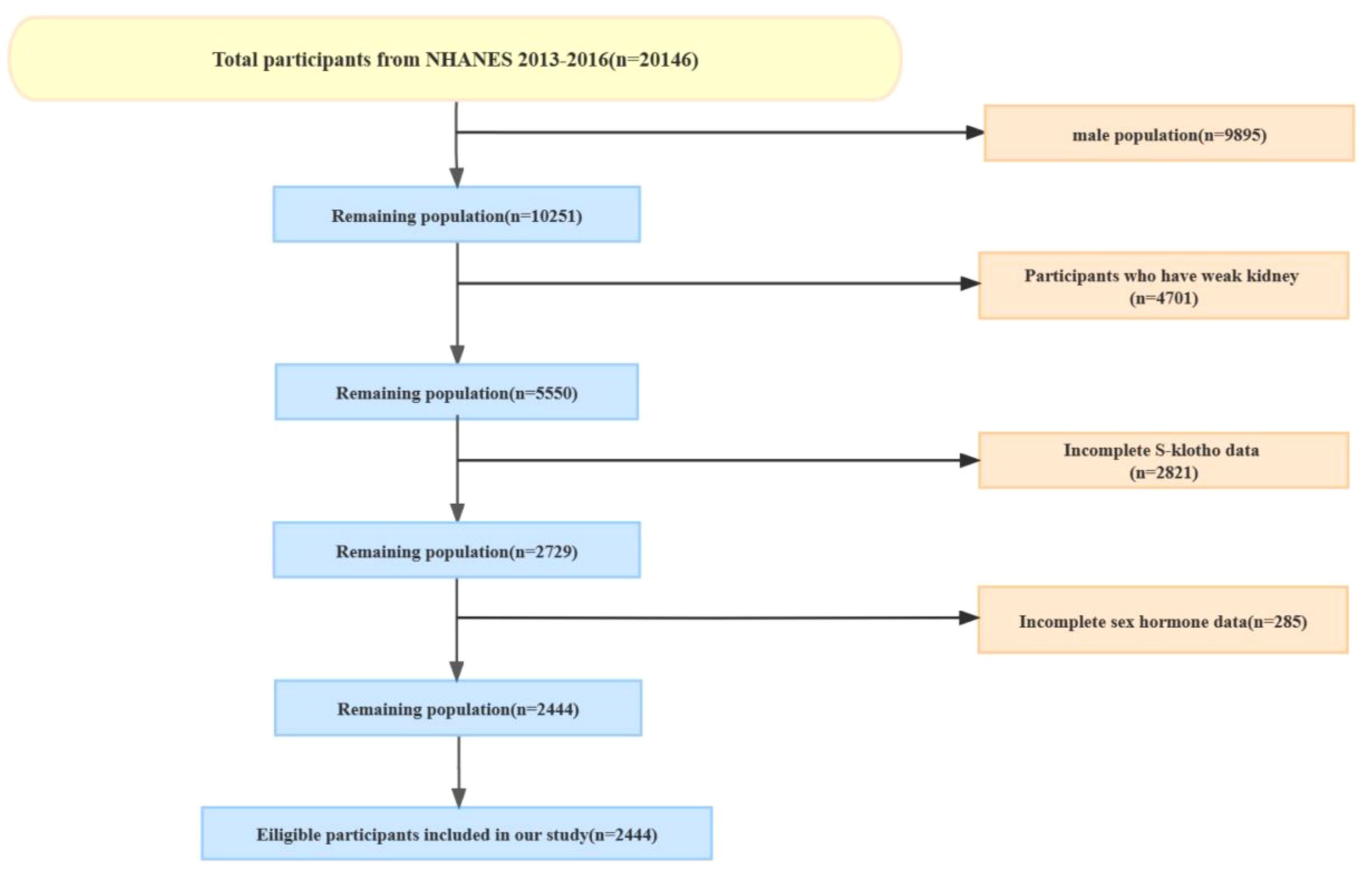
The participants enrollment.

This cross-sectional analysis sourced data from the National Health and Nutrition Examination Survey (NHANES), a recurrent nationwide survey overseen by the U.S. Centers for Disease Control and Prevention’s National Center for Health Statistics. NHANES evaluates the health and nutritional status of the U.S. populace in biennial cycles, employing representative sampling techniques. The NHANES protocol has received institutional review board approval, with participants granting written informed consent, aligning with the Declaration of Helsinki guidelines. The public can access this data via the CDC website: https://www.cdc.gov/nchs/nhanes/. We extracted data about sex hormones, SHBG, and S-klotho concentrations from NHANES 2013-2016. The cohort centered on female participants subjected to the mentioned tests. Female participants who completed the full 24-hour dietary history and underwent serum Klotho and sex hormone testing were included. Additionally, patients with renal failure were further excluded, as the major expression site of S-klotho is the distal convoluted tubules within the kidney (18) (**Figure 1**).

### Hormonal assays

Serum testosterone and estradiol were quantified using isotope dilution liquid chromatography tandem mass spectrometry (ID-LC-MS/MS), adhering to the National Institute for Standards and Technology’s (NIST) recommended method. SHBG measurements employed a two-step incubation process, reacting SHBG with immuno-antibodies and using a magnetic field. The resulting chemiluminescent reaction was recorded via a photomultiplier tube. Methodological specifics are available on the NHANES website. Additionally, the free androgen index (FAI) was computed using a formula integrating total testosterone and SHBG values, serving as a proxy for circulating free testosterone levels (14).

### S-klotho measurements

NHANES participants aged 40-79 had their pristine serum samples assessed using the IBL ELISA method. With the exception of four samples, the University of Washington’s Northwest Lipid Metabolism and Diabetes Research Laboratories conducted the analyses. Each sample underwent dual analyses, adhering to the manufacturer’s guidelines, ensuring the results conformed to the laboratory’s quality criteria.

### Covariates

#### Dietary inflammatory index

NHANES’s 24-hour dietary recall data served as the foundation for the DII, with its validity vouched for by the Nutrition Methodology Working Group (19). This recall system captures participants’ food and drink consumption in the day leading up to the interview. The DII was determined following the approach detailed by Shivappa et al. (20). With a higher DII denoting a pro-inflammatory diet and a lower value indicating the opposite, our research employed 27 of the 45 food parameters accessible in NHANES.

#### Other covariates

The study also accounted for variables including age, BMI, racial/ethnic background, family income relative to poverty (PIR), marital status, tobacco use, and alcohol consumption. The NCHS’s classification system was employed for smoking categorization, while alcohol intake was discerned from participants’ yearly consumption patterns. BMI, derived from self-reported height and weight, was segmented into standard categories as underweight or healthy weight (<25 kg/m^2^), overweight (25-29.9 kg/m^2^), or obese (≥30 kg/m^2^) (21). PIR was categorized as low income (<1), middle income (1-4), or high income (≥4).

### Statistical analysis

We conducted the statistical analysis using R (version 3.5.3) and EmpowerStats (www.empowerstats.com; X&Y Solutions Inc.). Categorical variables were presented as percentages, while continuous variables were reported as means with 95% confidence intervals (CI). Potential outliers were identified using box plots and carefully reviewed to assess their validity. Valid outliers were retained in the analysis, while any spurious outliers were excluded. Missing data were replaced with the mean.

To analyze differences among the S-klotho quarters, survey-weighted linear regression was used for continuous variables, survey-weighted Chi-square test was used for categorical variables: survey-weighted percentage (95% CI), 95%CI was by fitting a logistic regression model and computing a Wald-type interval on the log-odds scale.

The association between sex hormone and serum Klotho concentrations was estimated using multivariable linear regression models including model 1 (nonadjusted model) minimally adjusted model 2 (only age categorical, Race, Time of venipuncture were adjusted), and fully adjusted model 3 (age categorical, race, time of venipuncture, BMI, income-poverty ratio, alcohol drinking, smoking, waist circumference, physical activity level and DII were adjusted).

## Results

Between 2013 and 2016, the NHANES study enrolled 20,146 participants. Of this cohort, 10,251 were females and 9,895 were males. Exclusions were made for 4,701 females presenting renal insufficiency, 2,821 females lacking S-klotho data, and 285 females with incomplete sex hormone metrics. Consequently, our analysis incorporated 2,444 participants. The participant selection process is depicted in **Figure 1**.

**Table 1** delineates the baseline attributes of participants, stratified by Klotho quartiles. Females in the highest Klotho quartile, relative to their counterparts in the lowest quartile, demonstrated significantly reduced waist circumference and BMI values, decreased smoking prevalence, and a higher likelihood of identifying as Non-Hispanic Black.

**Table 1:** Baseline characteristics of female participants in 2013–2016 NHANES.

	Klotho Q1	Klotho Q2	Klotho Q3	Klotho Q4	*P*
Klotho (pg/ml)	557.05 (548.60, 565.49)	735.47 (731.19, 739.76)	904.38 (899.21, 909.55)	1272.99 (1244.36, 1301.63)	<0.001
BMI (kg/m^2^)	30.83 (30.12, 31.53)	30.43 (29.51, 31.34)	29.79 (29.05, 30.53)	29.58 (28.74, 30.42)	0.019
Waist Circumference (cm)	102.03 (100.44, 103.62)	100.46 (98.43, 102.50)	99.28 (97.68, 100.88)	98.30 (96.57, 100.04)	0.003
Testosterone (ng/dL)	22.74 (18.54, 26.94)	19.85 (18.47, 21.23)	23.99 (18.58, 29.41)	22.13 (19.32, 24.93)	0.275
Estradiol (pg/mL)	34.94 (27.47, 42.41)	44.79 (34.40, 55.19)	44.30 (33.47, 55.14)	44.36 (33.12, 55.59)	0.416
SHBG (nmol/L)	70.33 (66.44, 74.23)	70.61 (64.57, 76.65)	72.40 (67.33, 77.46)	74.65 (69.57, 79.74)	0.506
DII	1.11 (0.86, 1.35)	1.16 (0.97, 1.36)	1.20 (0.96, 1.43)	0.97 (0.74, 1.19)	0.507
FAI	1.39 (1.19, 1.60)	1.33 (1.18, 1.48)	1.36 (1.14, 1.58)	1.23 (1.13, 1.33)	0.488
Age					0.004
<=50	27.14 (22.11, 32.84)	30.82 (25.51, 36.69)	36.31 (30.72, 42.31)	37.45 (31.59, 43.70)	
>50, <=60	28.40 (23.70, 33.62)	34.62 (29.73, 39.86)	28.85 (23.79, 34.49)	30.77 (25.23, 36.93)	
>60	44.46 (40.31, 48.68)	34.56 (30.73, 38.59)	34.84 (30.05, 39.96)	31.78 (26.76, 37.26)	
Race					<0.001
Mexican American	6.24 (4.20, 9.19)	6.91 (4.32, 10.89)	8.88 (6.13, 12.68)	6.86 (4.53, 10.27)	
Other Hispanic	4.54 (3.16, 6.48)	3.74 (2.67, 5.21)	5.39 (3.70, 7.79)	7.19 (4.58, 11.12)	
Non-Hispanic White	75.22 (69.38, 80.27)	73.31 (67.75, 78.21)	69.23 (61.88, 75.72)	62.63 (54.92, 69.74)	
Non-Hispanic Black	7.14 (4.85, 10.40)	7.95 (5.24, 11.90)	8.46 (5.70, 12.38)	15.27 (11.47, 20.04)	
Other Race	6.85 (4.79, 9.72)	8.09 (6.10, 10.66)	8.05 (5.62, 11.41)	8.05 (5.86, 10.98)	
Time of venipuncture					0.900
Morning	49.44 (44.53, 54.36)	51.19 (47.10, 55.25)	48.89 (42.52, 55.30)	50.35 (43.43, 57.25)	
Afternoon	38.06 (32.74, 43.69)	34.72 (31.16, 38.47)	38.32 (32.62, 44.35)	35.08 (29.62, 40.95)	
Evening	12.49 (9.41, 16.41)	14.09 (11.20, 17.58)	12.79 (9.84, 16.46)	14.58 (11.69, 18.03)	
Alcohol drinking	71.41 (63.56, 78.14)	69.58 (63.18, 75.31)	70.65 (64.74, 75.94)	62.96 (57.79, 67.84)	0.072
Smoking					0.049
Never	54.52 (50.61, 58.38)	57.24 (51.99, 62.32)	61.05 (54.34, 67.37)	66.37 (61.73, 70.72)	
Former	26.43 (22.20, 31.13)	25.49 (20.10, 31.75)	21.85 (16.55, 28.27)	21.44 (18.25, 25.01)	
Current	19.05 (15.70, 22.92)	17.27 (13.08, 22.47)	17.10 (13.13, 21.96)	12.19 (8.85, 16.55)	
Physical activity level					0.391
Low	73.00 (67.89, 77.57)	71.36 (66.28, 75.96)	75.78 (71.00, 79.99)	69.77 (62.24, 76.37)	
High	27.00 (22.43, 32.11)	28.64 (24.04, 33.72)	24.22 (20.01, 29.00)	30.23 (23.63, 37.76)	
Income-poverty ratio					0.436
Low income	10.11 (7.00, 14.40)	12.78 (9.57, 16.87)	12.76 (9.87, 16.35)	13.69 (10.61, 17.51)	
Middle income	46.45 (39.33, 53.71)	46.49 (40.32, 52.77)	51.12 (44.52, 57.69)	45.67 (40.05, 51.40)	
High income	43.44 (34.39, 52.94)	40.73 (34.14, 47.66)	36.11 (28.98, 43.92)	40.64 (34.17, 47.45)	
Education level					0.101
Less than high school	13.63 (10.64, 17.28)	11.25 (8.23, 15.20)	13.12 (10.13, 16.83)	14.51 (11.47, 18.19)	
High school or GED	24.23 (20.15, 28.84)	19.06 (14.48, 24.68)	19.89 (15.77, 24.78)	16.32 (12.11, 21.64)	
Above high school	62.14 (57.64, 66.44)	69.69 (62.68, 75.88)	66.99 (60.87, 72.58)	69.17 (63.97, 73.92)	
Marital status					0.271
Living with partner	62.99 (57.40, 68.26)	67.51 (61.82, 72.72)	60.60 (55.01, 65.92)	63.77 (59.29, 68.02)	
Living alone	37.01 (31.74, 42.60)	32.49 (27.28, 38.18)	39.40 (34.08, 44.99)	36.23 (31.98, 40.71)	

Ranges of Klotho quartiles were presented as Min–Max.

Income-poverty ratio was categorized as low income (<1), middle income (1-4), or high income (≥4).

For continuous variables: survey-weighted mean (95% CI), P-value was by survey-weighted linear regression (svyglm); for categorical variables: survey-weighted percentage (95% CI), P-value was by survey-weighted Chi-square test (svytable). 95%CI of % was by fitting a logistic regression model and computing a Wald-type interval on the log-odds scale.

NHANES, The National Health and Nutrition Examination Survey; BMI, Body mass index; SHBG, The sex hormone-binding globulin; DII, Dietary Inflammatory Index.

**Table 2** details the correlations between sex hormones and S-klotho concentrations among female participants from the 2013-2016 NHANES cohort. In the unadjusted Model 1, a notable positive correlation was discerned between E2 and S-klotho concentrations (β 0.14, 95% CI 0.05-0.23), while FAI exhibited a negative association with S-klotho levels (β -7.08, 95% CI -13.39- -0.76). In the partially adjusted Model 2, SHBG was positively associated with S-klotho (β 0.43, 95% CI 0.06-0.80), and FAI maintained its negative relationship with S-klotho (β -9.73, 95% CI -16.6- -2.84). Upon full adjustment in Model 3, T (β 0.09; 95% CI -0.034-0.51), E2 (β -0.10; 95% CI -0.24-0.03), and SHBG (β 0.19; 95% CI -0.21-0.60) failed to show significant associations with S-klotho concentrations. It is worth highlighting that FAI’s negative relationship with S-klotho persisted, as evidenced by a coefficient of β -7.63 (95% CI -14.75-0.51). Due to the comparatively lower testosterone levels in females, the assessment of androgen levels may be more precise using the FAI rather than testosterone (22). Consequently, our results suggest a potential negative correlation between androgens (represented by FAI) and S-klotho.

**Table 2:** The association between sex hormones and S-Klotho concentrations among females in NHANES 2013-2016.

	*β (95%CI)*		
	Model 1	Model 2	Model 3
T, ng/dL	0.18 (-0.24, 0.60)	0.13 (-0.31, 0.57)	0.09 (-0.34, 0.51)
E2, pg/mL	0.14 (0.05, 0.23)*	0.06 (-0.07, 0.18)	-0.10 (-0.24, 0.03)
SHBG, nmol/L	0.35 (-0.03, 0.74)	0.43 (0.06, 0.80)*	0.19 (-0.21, 0.60)
FAI	-7.08 (-13.39, -0.76)*	-9.73 (-16.62, -2.84)*	-7.63 (-14.75, -0.51)*

NHANES, The National Health and Nutrition Examination Survey; CI, Confidence interval; T, testosterone; E2, Estradiol; SHBG, The sex hormone binding globulin; FAI, Free androgen index; DII, Dietary Inflammatory Index.

Model 1: unadjusted.

Model 2: partial adjusted by age categorical, race, time of venipuncture.

Model 3: fully adjusted by age categorical, race, time of venipuncture, BMI, income-poverty ratio, alcohol drinking, smoking, waist circumference, physical activity level, DII.

* means that there is significant ifference.

## Discussion

This cross-sectional study was conducted including a sample of 2,444 females in the U.S. Three models gradually adjusted for confounding factors, to help fully assess the associations between the indexes. This study verified that testosterone and estradiol levels seemed to have no association with S-klotho levels. Conversely, FAI exhibited a consistent negative correlation with S-klotho levels.

Sex hormones play active roles in the aging process in association with benign and malignant tumors of the breast and the reproductive system, as well as obesity (23, 24). They fluctuate across the menstrual cycle and consistently decline in the postmenstrual period with aging among females. Relatively low levels of sex hormones due to menopause are linked to altered inflammatory processes involving changing of cytokine levels and may cause immune senescence (25, 26).

Different from the result obtained from the male population (17), testosterone levels showed no association with S-klotho levels in our study. Combining to androgen physiology, the majority of androgens in females are precursors dehydroepiandrosterone (DHEA) and its sulfate, and androstenedione produced by the ovaries and adrenal cortices regulating by ACTH/LH axis (10). Testosterone production depends on direct ovarian secretion and androstenedione conversion in peripheral or extragonadal sites (10). After binding with DHEA producing by testosterone, the androgen receptor can be activated, and then lead to the activation of the *klotho* gene and subsequent expression of *klotho* mRNA in the distal convoluted tubules (27). The activation of the androgen receptor and subsequent activation of the *klotho* gene may be less significant in females due to much lower androgen levels, which maybe explain the limit to use testosterone as an independent index alone to assess the relationship with S-klotho in females. Further research is needed to fully understand the potential gender differences in the activation of the *klotho* gene by androgens.

High androgen status in women is supposed to be associated with inflammation, which is more accurate assessed by FAI than testosterone levels in the specific population with generally low-level testosterone. S-Klotho may activate NF-κB and suppress the production of pro-inflammatory cytokines (i.e., IL-6, IL-8, and TNF-α) to downregulate inflammation (28). This may explain the negative correlation between FAI and S-klotho levels in our findings to some extent. Therefore, it seems possible that FAI could predict inflammatory and aging state in females.

An earlier study posited a complex interrelation between estradiol levels and klotho gene expression, contingent upon specific renal contexts (16). However, this comparison might be incongruous as that study focused on mice and used uterine and kidney samples, whereas our study examined serum samples from female participants.

SHBG binds to approximately 44%-60% of the total serum testosterone. It’s pertinent to highlight that SHBG concentration influences the quantum of free or bioavailable testosterone, which has profound physiological implications. Previous literature has indicated that SHBG, rather than testosterone, might be a crucial metric associated with diabetes and metabolic syndrome, positioning SHBG as a viable metabolic risk biomarker (29). Our findings echo this perspective, demonstrating a more pronounced association of SHBG with S-klotho in females than testosterone, especially in Model 2.

This study’s robustness is attributed to its sizable cohort and gender-specific focus, lending credence to its generalizability. Nonetheless, there are intrinsic limitations. Solely measuring serum indices at one instance may not yield a holistic representation, and multiple measurements over time could enhance accuracy. Furthermore, the cross-sectional nature precludes establishing causality.

## Conclusions

Our study discerned a pronounced negative association between FAI and S-klotho levels, while testosterone and estradiol seemed to have a negligible impact on S-klotho concentrations in U.S. females. These insights elucidate the applicability of our findings to a female demographic and could potentially illuminate avenues for subsequent research endeavors.

## Data availability statement

The original contributions presented in the study are included in the article/supplementary material. Further inquiries can be directed to the corresponding authors.

## Ethics statement

The investigation was conducted as a cross-sectional analysis utilizing the NHANES database. The studies were conducted in accordance with the local legislation and institutional requirements. The investigation was conducted as a cross-sectional analysis utilizing the NHANES database. Written informed consent for participation was not required from the participants or the participants’ legal guardians/next of kin in accordance with the national legislation and institutional requirements.

## Author contributions

QC: Writing – original draft. JZ: Writing – original draft. XH: Writing – original draft. SD: Writing – original draft. LA: Writing – original draft. HZ: Writing – review & editing. WH: Writing – review & editing.
